# Neighborhood Exposures and Blood Pressure Outcomes: A Cross-Sectional Environmental Study among 19–53 Years-Old Parsis in Mumbai

**DOI:** 10.3390/ijerph18168594

**Published:** 2021-08-14

**Authors:** Hitakshi Sehgal, William A. Toscano

**Affiliations:** Division of Environmental Health Sciences, School of Public Health, University of Minnesota Minneapolis, Minneapolis, MN 55406, USA; tosca001@umn.edu

**Keywords:** neighborhoods, Parsi (Zoroastrian), environment, exposome, blood pressure, LMICs, Mumbai, India, urbanization, megacities, hypertension

## Abstract

The correlation between high blood pressure (BP) and urban neighborhood-level environmental determinants is understudied in low-income and middle-income countries (LMICs). We hypothesized that neighborhoods constitute exposures that affect resident-behaviors, metabolism and increased susceptibility to high BP. We studied urban clusters of Mumbai-Parsis (Zoroastrians), a founder population group, to minimize genetic variation and maximize exposure assessment. Participants from four neighborhoods were 19–53 years old and comprised 756 females and 774 males. We recorded healthy BPs (≤120/80 mmHg) in 59%, pre-hypertensive (≥121–139/81–89 mmHg) in 21% and high BP (≥140/90 mmHg) in 21% of the participants. A family history of hypertension had no correlation with high BP. We used the Neighborhood Accessibility Framework to compile a questionnaire in order to collect data on participants’ perception of space, third places, streetscape and experience, land use, connectivity, surveillance, pedestrian safety and public transport. Our results suggested that participants in neighborhoods with poorer BP outcomes reported lower accessibility scores for space, streetscape and experience, third places and connectivity. Our study evaluates how neighborhood-level determinants affect BP outcomes in order to contribute to the body of knowledge on primary preventive measures for high BP in urban LMIC populations. We concluded that neighborhood exposures affect resident-behaviors, which cause metabolic changes and increase susceptibility to high BP.

## 1. Introduction

As exposomes, neighborhoods are the interface between the micro-exposome of the home and the macro-exposome of city. Based on the scales of settlement hierarchy, we propose that the home is a micro-exposome, the neighborhood is an intermediate level exposome and the city beyond the neighborhood is the macro-exposome. The exposome includes every exposure outside the genome and, therefore, is a broad aggregate of (a) general external, (b) specific external and (c) internal exposures [1]. In the recent years, the exposome has been studied in relationship to parameters such as nutrition, urban living, fetal and developmental environments, low birth weight and psycho-socio-cultural factors [1,2]. The neighborhood lies at the nexus of an interconnected system of exposomes within the macro-urban exposome, and thus both local-level urbanization and macro-level globalization developments impact the neighborhood-level determinants [3].

Neighborhoods comprise intermediate level exposures, which include natural, built, economic and socio-cultural environments [4]. These exposures at the neighborhood-level influence resident-behaviors [4,5]. Neighborhoods affect physical activity, tobacco use, alcohol use, diet, street violence, safety, social capital, social cohesion, collective efficacy and sense of community [6,7], all of which determine health and disease outcomes. No single element is solely responsible for the adaptative and cumulative responses to neighborhood-level exposures [4,8]. Gaps in studies exist in the articulation of pathways, which link the neighborhood as an exposome to a common complex disease, such as high blood pressure (BP).

High blood pressure, the most significant risk factor for mortality from cardiovascular diseases (CVDs) [9], accounts for nineteen percent of global deaths [10]. Morbidity from high BP accounts for seven percent of all disability-adjusted life years lost [11]. Dubbed as the silent killer [10], high BP is often asymptomatic but may manifest as a headache, dizziness or nosebleeds [12]. High blood pressure is defined as BP raised over 140/90 mmHg [9,10]. A diagnosis of clinical hypertension is made when high BP (≥140/90 mmHg) is recorded on two or more consecutive visits to the clinic [13]. Hypertension often remains undiagnosed because BP is not routinely measured at clinics. Vascular damage and cardio-vascular events afflict people who have high BP but are not diagnosed with hypertension [10,12]. Therefore, higher than normal BP is classified as a health-risk [14]. People who experience high BP on occasion, either as white-coat hypertension or an incidental high BP reading, should be made aware of the disease and its risks [14].

Blood pressure regulation involves a complex interaction between specific single nucleotide polymorphisms (SNPs), genes and the exposome [10,15,16]. This implies that people who inhabit an unfavorable exposome are more prone to phenotypic expression of high BP than those who live in a health-promoting exposome [17]. A singular cause cannot be attributed to a common complex disease such as high BP, which is expressed as a consequence of reciprocity between internal and external factors, which are heritable SNPs and the exposome, respectively [18]. Globally, high BP correlates with heritable, sociodemographic, behavioral and metabolic risk factors across diverse ethnic populations [8]. In adverse exposomes, SNPs associated with BP result in the phenotypic expression of high BP [8]. Adverse conditions could arise in either the external or internal exposome or independently in both. Epigenetic research has put to rest the monogenic and singularly genetic view on factors that affect BP [19]. A range of environmental factors from maternal water deprivation and protein deficiency to hazardous exposures including pesticides and noise are reported to affect BP [19,20]. Studies suggest that physically active populations with low-sodium intake are the least likely to develop high BP [2,21]. Through its interaction with genetics, ageing and sex, the exposome is a principal and modifiable determinant of BP, but its role in affecting BP outcomes remains underexplored. In the last four decades, the prevalence of hypertension has increased exponentially in urban LMIC populations [20,22]. At a global level, three out of four hypertensives reside in LMICs [10,11,20]. The reported disproportionate increase in hypertension prevalence in LMICs [10,11,20] suggests heterogeneities in the urban exposome because the genome, sex and ageing are stable and predictable factors.

In urban India, the prevalence of hypertension has increased thirtyfold in a span of twenty-five years [20]. Urbanization, which is a known risk factor for high BP [12,22], is one of the pathways through which global environmental changes have a detrimental effect on the health of inhabitants [23]. Ageing and healthcare disparities do not adequately explain the disproportionate rise in high BP and other noncommunicable diseases (NCDs) in LMIC populations [24]. Healthcare initiatives, such as routine BP monitoring, are targeted more toward high-risk groups and only implemented after behavioral risk factors affect people [24,25]. Urbanization poses a primordial risk [25] because it escalates the probability of exposure to the first-level of risk factors. Therefore, changes to urban determinants offer a greater scope for the implementation of high BP prevention strategies. Urban living drives behavioral factors that result in metabolic changes and precipitate high BP [12,24]. There are gaps in understanding how this behavioral-metabolic pathway correlates with the neighborhood-level exposome, where the neighborhood is an intermediate link between the micro-exposome of the home and the macro-exposome of the city. Our aim was to conduct a population study to evaluate how neighborhood-level determinants affect BP outcomes, and thus contribute to knowledge on primary preventive measures for high BP in urban LMIC populations. Neighborhood affluence has been demonstrated as a greater predictor of health status than individual demographics, SES and health behaviors [26]. Rather than individual behaviors and socio-economic situations, we studied proximal determinants, which can guide preventive strategies for the continued increase in prevalence of high BP. Research and guidelines on high BP raise the concern that initiatives to control high BP and CVD are not universally successful [27]. Critical population-based initiatives need to be applied to reduce the burden of high BP [27]. Research on individual level factors that affect BP helps high-risk individuals but discounts the most critical question; i.e., why is BP high in some populations [28]? In this study, we tested the hypothesis that neighborhoods constitute exposures, which affect population behaviors and lifestyles, and thus result in metabolic changes that increase susceptibility to high BP. This research was a quantitative, cross-sectional and environmental epidemiological study. To the best of our knowledge, this study is the first of its kind in urban India to assess this microcosmic correlation between neighborhood-level exposures and high BP. 

## 2. Materials and Methods

We identified four typologies of Parsi neighborhoods in the city listed in Table 1 as the spatial units of exposome analyses; a founder population that resides in these four types of neighborhoods; a Neighborhood Accessibility Framework, which comprises natural, built and socio-cultural neighborhood-level determinants; and potential confounders of high BP. We collected the data by using a questionnaire and manually recorded height, weight and BP of the participants. 

Cohorts: Mumbai is a megacity with an ethnically and genetically heterogenous population. A cross-sectional study across the entire urban population would invariably include ethnic and genetic variations in high BP prevalence. Parsis are a founder population who largely reside in endogamous urban clusters but are not isolated from the city at large. A founder population has low genetic heterogeneity [29,30], which allowed us to examine the effect of neighborhood-level exposures on BP. In order to compare neighborhood types as exposomes, we classified Parsi neighborhoods into four typologies on the basis of key built-environment attributes, as listed in Table 1. Parsis are a community with no extreme poverty and higher standards of living with a history of exemplary social reform and attainment of education [31,32]. All participants in the study were educated above higher secondary school. This standardized socio-demographic background supported the aim to study the population rather than individual factors affecting the blood pressure of participants.

We classified Parsi urban clusters in Mumbai into four neighborhood typologies: Baugs, Parsi Apartments, Cosmopolitan Apartments and Parsi Colonies, as listed in Table 1. A Parsi Baug (coded as BAUG) is a gated community with the amenities such as a playground and a gymkhana. Gymkhana, which originates from Hindi or Persian language, is an enclosed space adjacent to a playground. Gymkhanas provide spaces for socializing, indoor games and may serve food. Parsi Apartments (coded as PARAP) could either be a cluster of several apartment buildings or an individual apartment building. We defined a Parsi Colony as non-gated community housing with the amenities of a playground and a gymkhana. The Mancherji Joshi Dadar Parsee Colony (coded as MJDPC) is the only non-gated Parsi housing with a gymkhana and a playground. All Baugs and apartments are conceptually and spatially similar, but they are not architectural replicas. The four cohorts included ten Baugs, forty-one Parsi Apartment clusters, seventy-nine Cosmopolitan Apartments and the Parsi Colony, MJDPC, which is one of a kind.

The sample size was 1530, which comprised 774 female and 756 male participants. The population of Mumbai is over 18 million. We calculated the sample size by using the formula for a large population: (Z-score)^2^ × Std Dev × (1 − Std Dev)/(margin of error)^2^. The Z-score for 95% CI (Confidence Interval) was1.96, where the standard deviation was 0.5 and the margin of error was 0.5. The minimum sample size was the following: minimum sample size = (1.96)^2^ × 0.5(1 − 0.5)/(0.5)^2^ = 370.

**Figure 1:**
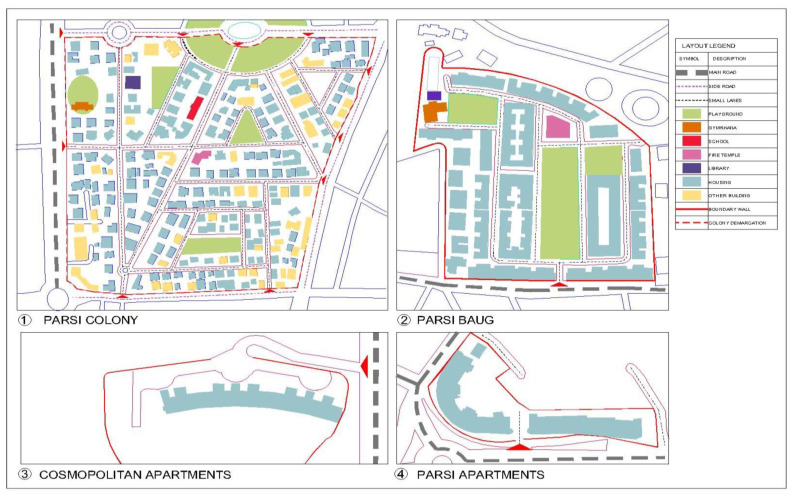
Schematic representation of neighborhood typologies (A schematic of the neighborhood typologies. The figure was made by drafting maps of the neighborhoods using the AutoCad 2017 Software).

We studied twenty variables including confounders that affect BP and factors that constituted the neighborhood. We multiplied this by ten and, thus, needed a minimum of two hundred participants from each neighborhood. Baugs and Parsi apartments have a greater population, and hence we recruited more participants from these neighborhoods. A random sample was secured from Parsi Baugs, Parsi Apartments and Parsi Colony as a house-listing was available. For cosmopolitan neighborhoods, we conducted snowball sampling because very few Parsis reside in cosmopolitan neighborhoods and are spread across the city. The large sample size mitigated bias, if any, that may have arisen due to the snowball sampling. Participants were recruited with the help of community leaders and volunteers. The features of the neighborhoods are described in Table 1 and depicted in Figure 1. Schematic representation of the neighborhoods was drawn by using the AutoCad 2017 Software (Autodesk Inc., San Rafael, CA, USA). 

Neighborhood, the independent variable, was the exposure, as listed in Table 1. Blood Pressure, the dependent variable as listed in Table 2 [12], was the primary outcome. 

We used the Qualtrics software, Version 2017 (Qualtrics, Provo, UT, USA, copyright © (2017) Qualtrics), to prepare the self-administered questionnaire and collected data, without identifiers, on a digital device (Samsung Galaxy 4 tablet, Samsung Electronics, Suwon-si, Korea). Please see Supplementary Materials for the Questionnaire. The questions for neighborhood perceptions were validated on the basis of published studies [7,33,34,35], interviews with Parsis and feedback from researchers. In order to determine whether the questionnaire effectively captured data on demographics, confounders for high BP and cumulative neighborhood-level determinants, we compiled the questionnaire from validated instruments. 

This study relies on the definition of accessibility as the ease with which individuals are able to fulfil their daily needs and partake in activities such as socializing, exercise and shopping in their immediate environment, which in this case is the neighborhood [36,37]. In order to measure accessibility through residents’ perception, we used the Neighborhood Accessibility Framework based on the Walkability Framework. To promote physical activity, the Walkability Framework theorizes the relationship between walkability and nine attributes of the built-environment: (1) connectivity, (2) density, (3) land use, (4) traffic safety, (5) parking, (6) surveillance, (7) experience, (8) greenspace and (9) community [35]. Our assessment was not restricted to walkability, and the Neighborhood Accessibility Framework provides a larger scope compared to the Walkability Framework. We have added the factors of space and third places and expanded the experience category into streetscape and experience. We retained the remaining categories in Walkability Framework: land use, connectivity, surveillance, pedestrian safety and public transport. We excluded the two categories of parking and density in the Neighborhood Accessibility Framework. The parking category measures the availability of parking space, which is in short supply in Mumbai. Therefore, participants’ perceptions would likely have been skewed based on their need to park their vehicle, rather than how parking sociospatially affected the neighborhood streetscape and space. Density is considered a positive influence on sociospatiality based on research on medium-sized cities [38]. Mumbai is an overcrowded megacity [39] and is, therefore, not comparable to results from medium-sized cities. We modified connectivity because the questions regarding block size were too technical for an average participant. We evaluated connectivity by using a visual analysis of neighborhood images, structural constructs, neighborhood layouts and the prevalence of mixed land use. We assessed participants’ perception of pedestrian paths within their neighborhood that facilitated access to walkable destinations. 

We repeated validation drills, which resulted in iterations of the questionnaire with fine tuning based on experts’ feedback until we developed a satisfactory questionnaire that had face and content validity. For methodological validation, we revalidated the cumulative responses of the participants using the Walk Score^®^, (https://www.walkscore.com/cities-and-neighborhoods/, accessed on 15 June 2017) both before and after data collection. The Walk Score is a peer-reviewed concept used in urban studies for planning and public health. Prior to data collection, we randomly surveyed people from the neighborhoods under study. The perceptions of the participants matched the Walk Score of each neighborhood, which revalidated our questionnaire. 

The first question constituted an informed consent, and the participants could only proceed with the questionnaire if they authorized us to record their data. The first section comprised questions on their home address; work location and mode of commute; age; gender; marital status; household income; family size; exercise frequency and duration; six or more sedentary hours spent per day; proxy evaluation of salt intake through number of homemade versus non-homemade meals (breakfast, mid-morning snack, lunch, evening snack and dinner), salt sprinkled on cooked meals and packaged beverages; smoking; alcohol use; excessive stress; a family history of hypertension and a self-reported history of diabetes, hypertension and antihypertensives. 

The second section assessed the participants’ perceptions of their neighborhood as an intermediate level exposome. We defined the neighborhood as a one-kilometer radial walkable precinct around the participants’ respective homes [34]. We compiled data on participants’ perceptions of their neighborhood into the Neighborhood Accessibility Framework, as shown in Table 3. The Framework, a cumulative matrix of neighborhood-level determinants included the following: space, third places, streetscape and experience, land use, connectivity, surveillance, pedestrian safety and public transport. This section had thirty-one questions associated with the built-environment and its sociospatial components. 

The questions in each category of the Neighborhood Accessibility Framework are abridged here. Each category of the framework comprised questions that recorded participants’ perceptions of accessibility. (1) Space: We define this is as a neighborhood space for physical activities and sports, including a community gymnasium and a swimming pool. (2) Third places: These are local gathering places within residential walkable radius without automobile dependency, which provide an alternative to digital entertainment [40]. For this study, we evaluated third places in terms of their (a) efficacy to facilitate opportunities that promote knowing and socializing with one’s neighbors and (b) access to venues for cultural activities (theater, art and social engagement), religious sites, educational facilities (public libraries and schools), community organizations and children’s park. (3) Streetscape and Experience: This category includes the urban design attributes of streets in terms of cleanliness, aesthetics and shade-providing trees, all of which promote sociospatiality. (4) Land Use: This comprised the extent of mixed land use for residential, retail and healthcare purposes in walkable proximity to the neighborhood. We used this category to assess ease of walkable access to (a) retail facilities for fresh food, meat and fish, groceries, a corner convenience store and a department store and (b) healthcare facilities such as a primary healthcare clinic, a 24 h pharmacy and a hospital with emergency care. (5) Connectivity: We evaluated the network of pedestrian paths to determine ease of destination access within the neighborhoods. (6) Surveillance: We defined this in terms of Jane Jacobs’ concept of “eyes on the street” and assessed this as perceptions of safety from crime based on gender, age and socio-economic status [35,41]. (7) Pedestrian Safety: We assessed this in terms of crosswalks, traffic lights and obstacle-free pedestrian paths within the neighborhood. (8) Public Transport: We defined this as access to public transport options for travelling to the larger urban area and the walkable radius of the neighborhood.

The affirmative responses to the questions in section two were summed up. We scored the percentage of affirmative responses for each question on a scale of 0–5. If over 90% of the participants from a neighborhood reported affirmative accessibility, then the score was 5; for 81–90% it was 4; for 71–80% it was 3; for 61–70% it was 2; for 51–60% it was 1; and responses below 50% resulted in a 0. Based on this scale, we calculated the affirmative response score for each question and for each neighborhood. We averaged the affirmative response scores in each category in the Neighborhood Accessibility Framework and scaled them from zero to ten in order to determine the neighborhood accessibility score, shown in Table 3. A score of zero meant that the resident-participants perceived a lack of accessibility, while a score of ten implied maximum accessibility. 

The third section includes measurements of participants’ (a) height, recorded on a stadiometer, (b) weight on a digital scale and (c) two BP readings, which were recorded manually using a sphygmomanometer. While it is recommended that average BP should be measured on several occasions, ours was a cross-sectional study that mimicked real-life situations where there are no resources to repeat BP readings at recommended intervals. We adopted a simplified BP measurement approach that minimized misclassification and can be used in resource poor settings [42]. Each BP reading was taken following a ten-minute rest. If the two readings of BP were more than 10% apart, the readings were repeated until we obtained two successive readings within close range. For analysis, an average of the two BP readings that resulted in a categorical variable was used. The first author collected the data, including BP measurement, within the neighborhoods and their respective community centers in a space allotted by the community members for conducting the study. Participants who recorded two readings of high BP were advised to consult with a physician if they had already not done so. No diagnosis of hypertension was made.

We distributed the age data statistically into quartiles, which resulted in four age-cohorts of the following: (1) 19–28 years, (2) 29–38 years, (3) 39–44 years and (4) 45–53 years. We created a smoking score that was quantified as follows: 0 = never smokers; 1 = non-daily smokers; 2 = past daily smokers; and 3 = current daily smokers. For exercise frequency and duration, we distributed the sample into categories of (1) inactive, (2) less than optimally active (<150 min) and (3) optimally active (≥150 min/week). We calculated the Body mass index (BMI) as weight in kilograms divided by height in meters squared. We categorized BMI outcomes as follows: (1) Underweight (<18.5), (2) Healthy (18.5–24.99), (3) Overweight (25–29.99) and (4) Obese (>30) (Source: Centers for Disease Control: https://www.cdc.gov/healthyweight/assessing/bmi/adult_bmi/index.html, last accessed on 28 April 2021).

Analysis: The independent variable had four categories (Table 1) and the dependent variable had three categories (Table 2), and therefore the use of multinomial regression analysis was required. Due to the large number of confounders, we performed this analysis in three models. We used the IBM SPSS Statistics for Macintosh, Version 25 software (IBM Corp., Armonk, NY, USA) for the analysis and conducted Chi-square tests, simple frequencies and crosstabs.

## 3. Results

Participants from the MJDPC had the most affirmative responses (80%) to the perceptions of neighborhood accessibility, followed by participants from BAUG (72%), COSMO (61%) and PARAP (59%). The mean length of stay in the current residence by neighborhood was (1) BAUG 23 years (SD = 11.95), (2) COSMO 21 years (SD = 13.76), (3) PARAP 20 years (SD = 13.41) and (4) MJDPC, 27 years (SD = 12.66). The Neighborhood Accessibility Framework (Table 3) includes accessibility factors and scores. 

Blood pressure outcomes for each age cohort are shown in Table 4.

Of all the participants, 5.8% (*n* = 89) reported current smoking use, 5.2% (*n* = 63) disclosed past daily smoking, 5.3% (*n* = 80) reported occasional smoking (past or present) and 82.4% (*n* = 1260) stated never having smoked. Alcohol use was prevalent in 8% (*n* = 128) of the participants. We recorded excessive stress in 37% (*n* = 564) of the participants. In the sample, 44% (*n* = 659) of the participants reported that they did not exercise routinely, while 56% (*n* = 856) reported that they exercised regularly. Of those who exercised consistently, 610 participants spent at least 150 min per week on exercise and 246 spent less than 150 min on exercise. The percentage of participants who reported sedentary habits was 50 (*n* = 771), but this showed no correlation with higher BPs in a chi-square analysis (prehypertension or high BP). Of the 1186 participants who traveled to work, 78% (*n* = 923) were automobile-dependent, 3% (*n* = 37) walked and 19% (*n* = 225) commuted by public transit, while 335 (22%) participants either worked at or from home.

Of the known hypertensives (*n* = 196), about half (*n* = 103) took antihypertensive medications, 47.4% (*n* = 93) continued to have high BP, 27.6% (*n* = 93) had prehypertension and 25% (*n* = 49) had controlled BP within normal limits. Of those who took antihypertensives, (*n* = 103), 19 (18.4%) of the participants had BP ≤ 120/80 mmHg, 34 (33%) presented with prehypertension and 50 (48.5%) had high BP. Of the participants with higher BPs, 44.6% (*n* = 279) did not exercise at all, 14.5% (*n* = 279) exercised <150 min/week [12], 51.4% (*n* = 322) had sedentary habits or deskbound work and 63.9% (*n* = 322) were automobile dependent for commutes to work. In a separate analysis, we found that both physical inactivity and automobile dependence affected obesity significantly. 

**Figure 2:**
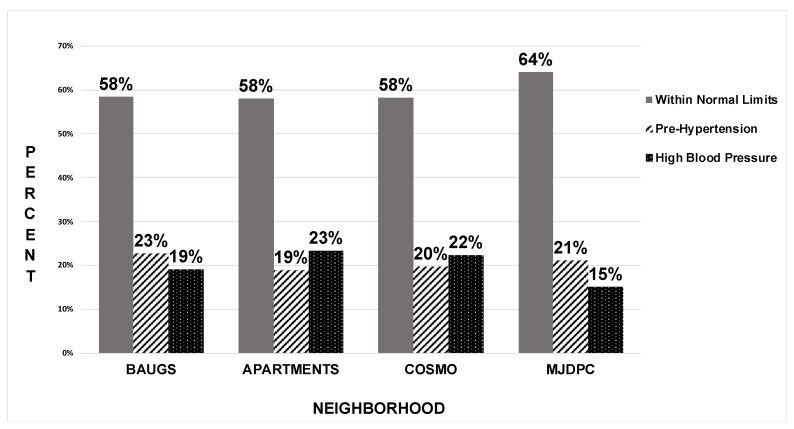
Blood Pressure prevalence among Parsis by neighborhood. Prevalence percentages of healthy, prehypertensive and high BPs in each neighborhood. The neighborhood cohorts are on the *X*-axis and BP prevalence percentages are on the *Y*-axis.

We documented healthy BPs in 903 (59%), pre-hypertensive BPs in 314 (20.5%) and high BPs in 313 (20.5%) of the participants. The prevalence of prehypertension in females was 13% (*n* = 99), which was lower than in males with 28% (*n* = 214), and high BP was equally distributed at 13% (*n* = 100) in females and 28% (*n* = 214) in males. The mean systolic BP of the population was 117 mmHg (SD = 16.56), and the mean diastolic BP was 78 mmHg (SD = 11.26). Figure 2 shows the BP prevalence distribution in the four neighborhoods.

The mean systolic BP of residents from the MJDPC was 115 mmHg; SD = 16.48, which was lower than those in COSMO (117 mmHg; SD = 16.51) and PARAP (118 mmHg; SD = 17.14). MJDPC residents had a lower mean diastolic BP (76 mmHg; SD = 12.36) compared to the cumulative and individual means in the other three neighborhoods.

The regression analysis was performed in three models. In models 1 and 2 of the regression analysis, we controlled the data for gender, age, BMI, family history and medications for hypertension, smoking, alcohol use, excessive stress, exercise frequency and duration, salt intake based on homemade and non-homemade meals. In model 3, we introduced the neighborhood variable. Table 5 (prehypertension) and Table 6 (high BP) show model 3 of the multinomial logistic regression analysis. The results show (Table 6) a significant association between neighborhoods and high BP prevalence. Residents of PARAP and COSMO were twice as likely to have high BP than those of MJDPC. 

We excluded participants with low BMIs (*n* = 47) because their numbers skewed the analysis, and therefore we compared the participants with healthy (40%, *n* = 604), overweight (35%, *n* = 539) and obese BMIs (22%, *n* = 340). Our data demonstrated that those with healthy BMIs (OR = 0.316, *p* < 0.05) and overweight BMIs (OR = 0.661, *p* < 0.05) were less likely to be pre-hypertensive, and those with healthy BMIs (OR = 0.098, *p* < 0.05) and overweight BMIs (OR = 279, *p* < 0.05) were less likely to have high BP compared to obese participants. Half of the participants (*n* = 765) reported a family history of hypertension. Those with no family history of hypertension were less likely (OR = 0.735, *p* < 0.05) to present with prehypertension. Family history had no significant (OR = 0.737, *p* > 0.05) impact on high BP. Known hypertensives were significantly likely to maintain pre-hypertensive (*p* < 0.05) and high BP (*p* < 0.05) levels. Smoking, alcohol habits and excessive stress did not affect BP outcomes. On an average day, 89% of participants ate one or more non-homemade meals. One non-homemade meal posed a lower risk (OR = 0.311, *p* < 0.05) for high BP over four non-homemade meals. Exercise frequency and duration did not significantly influence BP. 

## 4. Discussion

Our cross-sectional epidemiological examination of four distinct Parsi neighborhoods in Mumbai demonstrates that neighborhood-level exposures affected BP outcomes among residents. In neighborhoods with poorer BP outcomes, the participants perceived lower accessibility for streetscape and experience, third places, connectivity and space. BPs were high across the entire sample. 

Young Parsis presented signs of secondary or early ageing due to an unhealthy lifestyle. Participants older than 28 years had a greater risk of developing higher BPs, and 25% of participants ≤28 years presented with higher BPs. There is no defined year of life at which ageing results in the onset of high BP. Above 60 years of age [2], high BP prevalence is common in populations globally, and participants in our study were susceptible to higher BPs at a younger age by that measure. Our research supports the observation that in LMICs, urban exposomes result in an accumulation of behavioral risks at much younger ages, increasing the possibility of more people transitioning toward high BP [24] which, increases the lifetime risk of morbidity and mortality from CVDs.

The prevalence of high BP among young Parsi males (28%) and females (13%) was greater compared to adults (≥18 years) from the USA (males: 16% and females: 10.8%). Parsi males are at a greater risk for high BP compared to the general male population in India [22,43,44]. Premenopausal females have lower BPs [13], but the rising prevalence of higher BPs among young females is of concern. The increased prevalence of higher BPs (males: 56.6%; females: 25.6%) among Parsis was an important finding of our study. 

Measuring salt consumption is challenging, yet the evidence of its effect on BP outcomes among all other lifestyle influences is unmatched [2,20,45]. Those who frequently ate homemade meals (breakfast to dinner) were at equal risk of developing high BP compared to those who regularly ate (>four meals) non-homemade meals. This implied that homemade meals could have a high sodium content, which likely affected the participants’ BP. This could be from salt added during cooking. Studies have shown that in Asian cultures, higher sodium intake results from salt added during cooking [20]. 

The role of smoking, alcohol habits, stress and exercise cannot be ignored even if our results did not show correlations with higher BPs. Stress is difficult to quantify, and perhaps the participants underreported alcohol and tobacco consumption. Participants’ underreporting of tobacco use could have been due to the Parsi religious practice of fire-worship, which makes smoking a taboo among Parsis. 

Almost half the known hypertensives were not on antihypertensives. Only 49 known hypertensives had controlled their BPs (≤120/80 mmHg) with medication. Although this number of participants was small for statistical inferences, it indicates low levels of high BP control with medications and lifestyle changes. Among those with higher BPs, lower levels of physical activity and higher automobile dependence suggested unhealthy lifestyle choices. Lifestyle changes and therapeutics are important measures in secondary and tertiary public health outreach because controlled high BP could reduce the economic and healthcare burdens of CVDs [20]. The high rates of uncontrolled BP in LMICs underscores the need for public health interventions to remediate the lack of awareness, underscreening, undertreatment and poor control of high BP [10,20]. Participants who may have recorded high BP erratically due to white-coat hypertension or incidentally found their BP reading high should be made aware of their susceptibility to hypertension and its subsequent risks [14]. 

The association between heritability and BP is not well understood, and thus high BP fits the common complex disease paradigm [18]. In this Parsi population, which has a high prevalence (>20%) of high BP, modifiable environmental factors weighed over the unmodifiable factors of heritability to affect the phenotypic expression of high BP. Families share socio-cultural and behavioral environments that affect BP outcomes, and thus it is not possible to isolate these familial exposures from the genetic heritability of high BP. The BPs of young Parsis, a founder population, were comparable to BPs of the overall population of Mumbai [22,43,44]. In the assessment of the role of heritability, both these observations must be taken into account.

Research targeted at high BP treatment options related to the role of genetics, along with the 500 known SNPs associated with BP outcomes, is widespread [10]. However, research on the role of the epigenetic interaction with urban exposomes in both, BP outcomes and regulation remains underexplored, even though evidence suggests that genetics is only a permissive factor in response to environmental stimuli, as opposed to being a singular determinant of changes in BP [20]. The exposome is far-reaching, modifiable and presents underexplored opportunities in the prevention and control of higher BPs. The implementation of exposome modifications that can prevent, repair or even reverse epigenetic determinants offers possibilities to reduce the prevalence and strengthen the control of high BP. Since high BP is common at a global level, the diversity in exposomes may explain the variations in its prevalence across the world. It is premature to conclude that ethnicity alone accounts for regional-level variations in high BP prevalence until the diversity of the exposomes is rigorously examined.

Obesity is also a common complex disease that often coexists with hypertension and increases the risk for high BP [2,22]. Our results indicated a strong correlation of obesity with higher BP values. The exact mechanism by which obesity increases BP is as of yet unknown. The exposures associated with obesity comprise urbanization and improved standards of living that result in physical inactivity, coupled with greater food availability and choice [20,22]. South Asians are arguably more susceptible to obesogenic environments [22]. Our results challenge the widely held concept of a direct correlation between ethnicity and the common complex diseases of obesity and high BP. We observed high BPs in an ethnically exclusive Parsi community comparable to the prevalence of high BPs to other ethnic clusters in Mumbai. 

Residents of PARAP and COSMO reported poor accessibility scores for space and had poor BP outcomes. In BAUG and MJDPC the proximity of the playground and gymkhana resulted in higher accessibility scores for space, and their residents had better BP outcomes. In a megacity where people perceived a deficiency of space, access to amenities was a sign of neighborhood affluence, which can improve health irrespective of individual socio-economic status, behaviors and demographics [26]. 

When people stay longer in less affluent neighborhoods, residential stability is related to poor health, but better health outcomes are achieved when people live longer in affluent neighborhoods [26]. The residents of MJDPC had greater residential stability (27 years), but the average length of stay among all participants was 23 years. Thus, the length of stay was not significantly different among participants in the study.

Third places within neighborhoods are sites for sociospatial opportunities that facilitate neighborly interaction [46]. Third places are central to the Neighborhood Accessibility Framework. Third places enhance the social purpose and experience of being in a neighborhood because they reinforce other factors in the framework. They encourage access to destinations on foot and, consequently, improve surveillance, streetscape and land use. Thus, third places provide mobility and build social capital in the neighborhood, both of which positively influence BP. Other research has shown that the perception of social support correlates with health outcomes more than actual social support [26]. The low accessibility scores for third places in neighborhoods correlated with the BP outcomes. Accordingly, MJDPC residents reported higher accessibility scores for third places, while PARAP and COSMO recorded lower scores. 

In terms of neighborhood typologies, BAUG is closer to MJDPC than PARAP and COSMO, yet the mean BP of BAUG residents was comparable with those of PARAP and COSMO but higher than that of MJDPC residents. A gated community restricts access to the adjoining urban areas and provides no retail land use. This conceivably contributed to the relatively poor accessibility scores for third places, land use and connectivity among BAUG residents. The non-gated layout of MJDPC probably provided residents with more destination choices and mobility. 

The results of our study demonstrate that the effects of neighborhood-level determinants cannot be isolated [4]. Neighborhood-level determinants work collectively to constitute the neighborhood as an exposome that affects BP outcomes. For residents of MJDPC, no single determinant but the cumulative effect of more space, access to third places, better land-use and connectivity in the neighborhood resulted in participants’ perceptions of good surveillance and better accessibility within the neighborhood. In PARAP and COSMO, despite the positive perception of mixed land use and surveillance, the residents scored the remaining factors poorly. Neighborhoods that are at a structural disadvantage, in this case PARAP and COSMO, are shown to be less capable of maintaining social networks and subsequently experience lack of local social support and sociability [26]. The lack of neighborhood affluence as well as structural disadvantage manifested as limited space, third places, streetscape and experience and connectivity; therefore, residents of PARAP and COSMO had poor accessibility scores for third places, streetscape and experience, space and connectivity and were at a significantly (*p* < 0.05) greater risk of developing high BP compared with the residents of MJDPC. The perceptions captured restricted mobility that exacerbated the lack of accessibility to the built and socio-cultural neighborhood resources. 

Neighborhoods affected BP beyond their influence on active behaviors and BMIs. Exercise habits among young Parsi adults (56%) were higher than reported for urban Indians (8%) [47] but participants were largely automobile dependent for traveling to work. This is inconsistent behavior with their reported exercise habits in addition to their reasonable accessibility scores for land use, surveillance and public transport. This discrepancy between neighborhood accessibility scores and automobile dependence implies that other determinants mediated their unhealthy choice. The influence of other determinants is supported by the prevalence of high BP among residents of MJDPC (36%) and BAUG (41%).

Participants of all neighborhoods perceived poor pedestrian safety. India has an unenviable record of traffic injuries, which are the sixth leading cause of mortality in the country [48]. Inadequate implementation of urban planning polices results in low levels of pedestrian safety that increases the risk of pedestrian injuries, which likely discourages walking to contribute to automobile dependence [23] and sedentary behavior.

Studies from high-income countries (HICs) suggest that higher population density promotes walkability. The global livability index reports that all except two of the high-ranking cities of the world are mid-size cities [38]. Megacities in LMICs have high population densities but suffer overcrowding driven by migration, employment opportunities and climate change [23]. This discourages pedestrian activity and access to public transport, which presumably forces people to choose automobiles over walking. 

The ageing of populations, longer life expectancy and controlled infectious diseases are incomplete and simplistic explanations for the challenges posed by NCDs in LMICs [24]. Age, sex and genetics are non-modifiable factors that influence blood pressure [44]. Our study indicates early ageing among participants and higher BPs in both genders, which were affected by changes in socio-demographic attributes, reinforcing the role of the exposome as the modifiable influence on BP. Socio-demographic factors in LMIC populations contribute to a greater burden of high BP, and urban residents are at a higher risk than rural inhabitants within this LMIC distribution [20]. Macrosocial and macroeconomic determinants modify urban exposomes, which contribute to the increased prevalence of NCDs. Over the last three decades, India has rapidly urbanized and simultaneously recorded a significant surge in the prevalence of high BP and CVDs [9,20,22]. Mumbai has transitioned into a megacity and is the fourth most populous city in the world [49]. Urban configurations alter the built environment that affects the lifestyle choices available to people and their behaviors [50]. Changing behaviors cannot simply be assigned to personal choices when they are observed across populations. Rapid urbanization is associated with hypertensinogenic exposures such as sedentary life, increased salt intake, automobile dependence, obesity and physical inactivity [20,23]. Urbanization affects NCDs by social stratification [23], and we documented this through lack of third places, which affected the BP of participants in our study. Urban sprawl severed connectivity and encroached on spaces around PARAP and COSMO, while the MJDPC and BAUG could restrict sprawl in their immediate vicinity. Residents chose behaviors to adapt to the constraints of their neighborhood. These behaviors constituted lifestyle choices, which affected their metabolism and increased their susceptibility to high BP. Urbanization affects neighborhoods [23,24,50], and the extent of urbanization may determine the difference in the prevalence of high BP [20]. The decreasing order of scores of the Neighborhood Accessibility Framework implied that the extent of urbanization was in the order MJDPC, BAUG, COSMO and PARAP. With the loss of perception of every neighborhood determinant, the likelihood of developing a higher BP increased, even at a young age. The overall prevalence of higher BPs suggested that urbanization, a homogenous exposure, likely affected all neighborhoods, but neighborhood heterogeneity reflected the changing susceptibilities among participants. 

### Strengths and Limitations

Our study presented pathways by which cumulative assimilation of the natural, built and sociospatial attributes of a neighborhood affected the BP outcomes of the residents. To our knowledge, this is the only study in a LMIC megacity to present such a vast compilation of urban neighborhood data in correlation to high BP. Our results indicated early ageing in the population and placed into perspective the disproportionately high prevalence of high BP in LMIC urban areas. Our study articulated people’s perception of their neighborhoods and the consequent adoption of unhealthy lifestyles resulting in increased susceptibility to higher BPs. 

We did not evaluate some of the known factors that include pollution, early-life influences, placental insufficiency, maternal malnutrition, epigenetic effects and several social determinants, which are known to affect BP but were beyond the scope of this study. We posed two questions of particular interest, income levels and birth weight, for which we did not capture adequate number of responses. If the participants had provided these data, they would have provided us with greater insight into how income and birth weight affected BP. Of our sample, less than 30% of the participants were aware of their birth weight, and therefore available data were not enough for statistical analysis and comparison. While there is evidence that the Parsi population possess access to quality healthcare, household income data are important. Studies have found that socio-economic status affects control of hypertension by aiding healthcare and lifestyle modification [45]. Only 33% of the participants answered the question about their annual income. Of those who did answer the income question, some reported it as ‘zero’ and several participants informed us at the time of data collection that they would be unable to provide an accurate figure for their income. 

## 5. Conclusions

Preventive population strategies that will encourage people to adopt healthy behaviors and lifestyles are imperative for slowing the rapidly increasing prevalence of high BP in LMICs. In the realm of public health outreach, cumulative effects of neighborhood-level exposures can increase or decrease the risk of high BP. Our study provides an opportunity for strategizing prevention of high BP at the local level. The neighborhood lies at the cusp of the globalization and urbanization pathways that affect common complex diseases in populations. Addressing the complexities of high BP requires an interdisciplinary approach. Space, third places, streetscape and experience, land use, connectivity, surveillance, pedestrian safety and public transport are neighborhood-level determinants that affect accessibility and should be addressed in future urban development initiatives. Public health professionals and urban planners should work together to positively change urban design for better health behaviors and outcomes. The potential of migration and urbanization and the increasing prevalence of common complex diseases in LMICs calls for the inclusive planning of neighborhoods. When health will be a part of every policy, we will inch closer to the sustainable development goal of good health and well-being (goal 3).

## Figures and Tables

**Table 1:** Summary of four neighborhoods and participants.

Neighborhood Typology (Independent Variable)	Key Built-Environment Attributes	Age Cohorts				Gender		Total Participants
		1	2	3	4	F	M	
Parsi Baugs (BAUG)	Exclusive, gated, community housing with amenities	157	144	96	108	253	252	505 (33%)
Parsi Apartments (PARAP)	Exclusive community housing without amenities	134	139	96	136	263	242	505 (33%)
Cosmopolitan Apartments (COSMO)	Housing anywhere in the city with other ethnicities	51	90	84	81	155	151	306 (20%)
Parsi Colony (MJDPC)	Exclusive community housing with amenities, but not gated	61	46	50	57	103	111	214 (14%)

Inclusion criteria for the study were as follows: an exclusive Parsi lineage (all grandparents and parents were Parsi); spoken and written English proficiency; at least three years of residency in the current neighborhood; and ages between 19 and 53 years.

**Table 2:** Blood Pressure Measures and Categories.

Systolic	Diastolic	Category
≤120 mmHg	≤80 mmHg	Within Normal Limits
=121–139 mmHg	=81–89 mmHg	Pre-Hypertensive
≥140 mmHg	≥90 mmHg	High Blood Pressure

**Table 3:** Accessibility Framework: accessibility score based on participants’ perceptions of neighborhood.

Neighborhood Accessibility Framework					
	Accessibility Category	Accessibility Score			
		BAUG	PARAP	COSMO	MJDPC
1.	Space	6.4	1.6	2.4	8
2.	Third Places	5.5	2.8	1.8	8.3
3.	Streetscape and Experience	8	2	2.7	9.3
4.	Land Use	7.3	8.5	8.5	9.3
5.	Connectivity	4	2	2	8
6.	Surveillance	10	10	9.3	8
7.	Pedestrian Safety	2	0	0	5
8.	Public Transport	8	8	8	10

Accessibility Score: zero indicates the lack of accessibility, and ten indicates maximum accessibility.

**Table 4:** Blood pressure and age cohorts.

Blood Pressure	Age ^c^ Cohorts			
	19–28 Year	29–38 Year	39–44 Year	45–53 Year
**Within Normal Limits**	72.0 ^a^ (290) ^b^	60.4 (253)	55.2 (180)	47.4 (181)
**Pre-Hypertensive**	15.9 (64)	20.3 (*n* = 85)	21.2 (69)	24.9 (95)
**High Blood Pressure**	12.2 (49)	19.3 (81)	23.6 (77)	27.7 (106)

^a^ Values are percent; ^b^ number of Participants; ^c^ the mean age of participants was 36.5 years (SD = 9.72).

**Table 5:** Multinomial logistic regression for pre-hypertension and neighborhoods.

Prehypertension						
Multinomial Logistic Regression						
Model 3						
Variables		β	*p*	Odds Ratio (OR)	95% CI for Coefficient	
					Lower	Upper
Gender	Females	−1.530	0.000	0.216	0.158	0.297
	Males	Reference Category				
Age Cohort	19–28 years	−0.717	0.001	0.488	0.319	0.748
	29–38 years	−0.321	0.112	0.725	0.488	1.078
	39–44 years	−0.052	0.804	0.949	0.629	1.432
	45–53 years	Reference Category				
Body Mass Index Category	Healthy 18.5–24.99	−1.152	0.000	0.316	0.209	0.478
	Overweight 25–29.99	0.413	0.038	0.661	0.448	0.977
	Obese 30–49.99	Reference Category				
Family History of Hypertension	No	−0.308	0.040	0.735	0.548	0.986
	Yes	Reference Category				
Medications for Hypertension	No	−1.242	0.000	0.289	0.151	0.552
	Yes	Reference Category				
Smoking Score	Never	0.055	0.852	0.947	0.533	1.683
	Non-Daily	−0.157	0.706	0.855	0.379	1.931
	Past-Daily	−0.144	0.725	0.866	0.389	1.930
	Current-Daily	Reference Category				
Alcohol over three times/week	No	0.105	0.686	1.111	0.666	1.854
	Yes	Reference Category				
Excessive Stress in last six months	No	−0.003	0.984	0.997	0.737	1.349
	Yes	Reference Category				
Exercise duration	No	−0.139	0.397	0.870	0.631	1.200
	<150 min/week	−0.378	0.088	0.685	0.444	1.057
	>150 min/week	Reference Category				
Eat Non-Homemade Meals	None	−0.336	0.455	0.714	0.295	1.728
	1 Meal	0.432	0.336	0.649	0.269	1.565
	2 Meals	−0.411	0.372	0.663	0.269	1.634
	3 Meals	−0.373	0.472	0.689	0.250	1.899
	4 Meals	Reference Category				
Neighborhood	BAUG	0.164	0.469	1.178	0.756	1.835
	PARAP	−0.075	0.748	0.928	0.587	1.466
	COSMO	−0.095	0.709	0.910	0.554	1.494
	MJDPC	Reference Category				

Significant *p* values (*p* < 0.05) suggested, gender, age, BMI and a family history of hypertension affected prehypertension in this cohort.

**Table 6:** Multinomial logistic regression for high blood pressure and neighborhoods.

High Blood Pressure						
Multinomial Logistic Regression						
Model 3						
Variables		β	*p*	Odds Ratio (OR)	95% CI for Coefficient	
					Lower	Upper
Gender	Females	−1.615	0.000	0.199	0.141	0.280
	Males	Reference Category				
Age Cohort	19–28 years	−0.820	0.001	0.440	0.275	0.705
	29–38 years	−0.382	0.071	0.683	0.451	1.033
	39–44 years	0.118	0.589	0.889	0.580	1.363
	45–53 years	Reference Category				
Body Mass Index Category	Healthy 18.5–24.99	−2.324	0.000	0.098	0.064	0.151
	Overweight 25–29.99	−1.275	0.000	0.279	0.192	0.406
	Obese30–49.99	Reference Category				
Family History of Hypertension	No	−0.305	0.057	0.737	0.539	1.008
	Yes	Reference Category				
Medications for Hypertension	No	−1.463	0.000	00.231	0.122	0.439
	Yes	Reference Category				
Smoking Score	Never	0.166	0.614	1.181	0.619	2.251
	Non-Daily	0.060	0.896	0.942	0.384	2.312
	Past-Daily	0.000	1.000	1.000	0.416	2.405
	Current-Daily	Reference Category				
Alcohol over three times/week	No	0.048	0.864	1.050	0.602	1.830
	Yes	Reference Category				
Excessive Stress in last six months	No	−0.072	0.657	0.930	0.677	1.279
	Yes	Reference Category				
Exercise duration	No	0.174	0.328	1.190	0.840	1.685
	<150 min/week	0.059	0.800	1.061	0.671	1.677
	>150 min/week	Reference Category				
Eat Non-Homemade Meals	None	−0.596	0.169	0.551	0.236	1.287
	1 Meal	−1.167	0.007	0.311	0.133	0.731
	2 Meals	−0.756	0.088	0.470	0.197	1.120
	3 Meals	0.428	0.387	0.652	0.248	1.717
	4 Meals	Reference Category				
Neighborhood	BAUG	0.489	0.072	1.631	0.957	2.780
	PARAP	0.561	0.038	1.753	1.033	2.976
	COSMO	0.597	0.039	1.817	1.031	3.201
	MJDPC	Reference Category				

Significant *p* values (*p* < 0.05) suggested, gender, age, BMI and neighborhoods affected high blood pressure in this cohort.

## Data Availability

The informed consent form and any other supplementary data will be shared upon request.

## Supplementary Materials

The following are available online at https://www.mdpi.com/article/10.3390/ijerph18168594/s1, Questionnaire: Neighborhoods and Blood Pressure.
