# Supplementary material for: Neighborhood Exposures and Blood Pressure Outcomes: A Cross-Sectional Environmental Study among 19–53 Years-Old Parsis in Mumbai
Source: Int J Environ Res Public Health. 2021 Aug 14;18(16):8594. doi: 10.3390/ijerph18168594 (PMC8391786; doi:10.3390/ijerph18168594)
Supplement: Supplementary file 1 [file ijerph-18-08594-s001.zip › ijerph-1337090-supplementary.pdf]

Neighborhoods and Blood Pressure

Start of Block: Default Question Block

Q1 **I have read the informed consent provided to me and I am participating in this study. I have secured contact information for Dr. Hitakshi Sehgal and will speak/ contact with her in case I have any questions.** (Please be assured that all information is secure and will not be shared or used for any other purposes than this research. We are not seeking any identifying information.)

○ **Agree** (1)

○ **Disagree** (2)

Skip To: End of Survey If I have read the informed consent provided to me and I am participating in this study. I have s... = Disagree

---

Q2 The following questions are about: Location of your residence and office,Past residences Month and year of birth, gender and Marital status, family size and family income

---

Q3 Please provide your **current residential address with the area pin-code:** (without house or flat number).

○ Building *(Ex. Firoz Apts)* (1) ____

○ Area 1 (*Ex. Kala Ghoda*) (2) ____

○ Area 2 (*Ex. Byculla E*) (3) ____

○ Pincode (*example:400020*) (4) ____

Q4 For how many **years** have you resided in your current neighborhood?
(Please enter a number).
_______ Years (1)

Q5 Do you travel to work/ college daily?

- ○ Yes (1)
- ○ No (2)
- ○ Refuse to answer (3)

Skip To: Q8 If Do you travel to work/ college daily? = No

Skip To: Q8 If Do you travel to work/ college daily? = Refuse to answer

---

Q6 How do you travel to work/ college? (Choose multiple if you change transport during one trip. Otherwise choose the most likely way you travel)

- ☐ Bus or Train/ Metro (1)
- ☐ Cab/ Autorickshaw (2)
- ☐ Car (with driver) (3)
- ☐ Car (self-drive) (4)
- ☐ Walk (5)
- ☐ Motobike (6)

Q7 Please provide an address for your work/ college destination: (The purpose of this question is to understand your daily commute)

- ◯ Area 1 (*Ex. Sakinaka*) (1) ____
- ◯ Area 2 (*Andheri East*) (2) ____
- ◯ Pincode (*example 400011)* (3) ____

Q8 Have you ever lived in any other neighborhood/s?

- ◯ Yes (1)
- ◯ No (2)
- ◯ Refuse to answer (3)

Skip To: Q12 If Have you ever lived in any other neighborhood/s? = No

Skip To: Q12 If Have you ever lived in any other neighborhood/s? = Refuse to answer

Q9 Have you lived in a Parsi Baug in the past?

- ◯ Yes (1)
- ◯ No (2)

Skip To: Q12 If Have you lived in a Parsi Baug in the past? = No

Q10 Please provide the name of the Baug and duration of residence.

	Past Neighbourhoods	Number of years of residence

	Name of the Baug (1)	Years (1)
1. (1)		
2. (2)		
3 (3)		

Q11 Please indicate your marital status:

- ☐ Never married (1)
- ☐ Separated (2)
- ☐ Divorced (3)
- ☐ Married (4)
- ☐ Widowed (5)
- ☐ Refuse to answer (6)

Q12 How many people currently live in your house (including yourself)?

________ Please enter a number (1)

Q13 What is the **annual income** of your household? (household income: income of all working members in your home)
If the annual income of your household is 5 lakhs, please enter '5' in the space below.

○ Annual Income (*do not write the zeros in the lakhs*) (1)
____

○ Don't Know (2)

○ Refuse to answer (3)

Q14 Please indicate your gender:

○ Female (1)

○ Male (2)

○ Other (3)

○ Refuse to answer (4)

JS

Q15 Please enter your birth month and year:

	Month	Year
Please Select: (1)	▼ 1 (1 ... 12 (12)	▼ 1900 (1 ... 2049 (150)

Q16 Birthday Reformatted

____

End of Block: Default Question Block

Start of Block: This section has questions on physical activity

Q17 This section has questions about physical activity.

Q18 **Do you regularly engage in a fitness activity?** (brisk walking for exercise/ jogging/ gym/ pilates/ power yoga/ play a sport/ other)
(*Regular = more than thrice a week*)

- Yes (1)
- No (2)
- Refuse to answer (3)

Skip To: Q22 If Do you regularly engage in a fitness activity? (brisk walking for exercise/ jogging/ gym/ pilate... = No

Skip To: Q22 If Do you regularly engage in a fitness activity? (brisk walking for exercise/ jogging/ gym/ pilate... = Refuse to answer

Q19 Please name the activity/activities:

- ☐ Activity 1 (1) ____
- ☐ Activity 2 (2) ____
- ☐ Activity 3 (3) ____

Q20 On a regular day, how many minutes do you spend on fitness?
(Please enter a number)
_______ Minutes (1)

Q21 On an average, how many days a week do you follow this fitness regime?
________ Days/ week (1)

Q22 Do you have a sedentary lifestyle? i.e. Do you/ Must you spend most of your day sitting? **(more than six hours)**

- ○ Yes (1)
- ○ No (2)

End of Block: This section has questions on physical activity

Start of Block: This section has questions on how much salt you consume on a regular day

Q23 **The following questions are about the nature of your meals to assess their salt content**

Q24 Please see the matrix below. On a regular day, do you eat homemade meals? Do you tend to skip some meals?
(Regular: more than five days/ week)

	Homemade (1)	Not homemade (2)	Skip (3)
Breakfast (1)	○	○	○
Mid morning snack (2)	○	○	○
Lunch (3)	○	○	○
Evening snack (4)	○	○	○
Dinner (5)	○	○	○

Q25 **For your homemade meals, are packaged foods used for cooking?**
(examples: ready cereals/ frozen cooked food/canned vegetables/ sausages/ salami/ frozen cooked meat/ instant noodles/ instant pasta/ other instant food mixes)

○ Yes (1)

○ No (2)

Q26 **For your homemade meals, are condiments like packaged cheese/ mayonnaise/ ketchup/ packaged salad dressings/ chinese sauces used?**

○ Yes (1)

○ No (2)

Q27 Do you regularly eat pickles (achaar) with homemade meals? *(Regular: with more than three meals a week)*

- ○ Yes (1)
- ○ No (2)

Q28 On a regular day, do you consume papad/ salli/ chips/ wafers/ chivda/ chaklee/ any other packaged salted fritters? *(Regular: More than thrice a week)*

- ○ Yes (1)
- ○ No (2)

Q29 Do you **regularly** consume **packaged beverages** (example: colas/ fruit juices/ other packaged drinks)? (Regular: More than thrice a week)

- ○ Yes (1)
- ○ No (2)

Q30 Do you find yourself occasionally adding extra salt to your food?

- ○ Yes (1)
- ○ No (2)

End of Block: This section has questions on how much salt you consume on a regular day

---

Start of Block: This section has questions on your exposure to smoke from tobacco or other drugs

Q31 **The following questions assess your exposure to first or secondhand tobacco smoke.**

Q32 Are you a smoker?

- Yes (1)
- No (2)
- Refuse to answer (3)

Skip To: Q35 If Are you a smoker? = No
Skip To: Q37 If Are you a smoker? = Refuse to answer

Q33 How often do you smoke?

- Daily (1)
- Not Daily (2)

Skip To: Q37 If How often do you smoke? = Daily
Skip To: Q34 If How often do you smoke? = Not Daily

Q34 Did you smoke **daily in the past**?

- Yes (1)
- No (2)
- Refuse to answer (3)

Skip To: Q37 If Did you smoke daily in the past? = No
Skip To: Q37 If Did you smoke daily in the past? = Yes
Skip To: Q37 If Did you smoke daily in the past? = Refuse to answer

Q35 Have you ever been a smoker in the past?

○ Yes (1)

○ No (2)

○ Refuse to answer (3)

Skip To: Q37 If Have you ever been a smoker in the past? = No

Skip To: Q37 If Have you ever been a smoker in the past? = Refuse to answer

---

Q36 How often did you smoke in the past?

○ Daily (1)

○ Not Daily (2)

Q37 Have you had long-term exposure to second hand tobacco smoke? **i.e. has anyone you have lived with, worked with, or have spent much time with, been a smoker?** *(Parent/ sibling/ close family member/ spouse/ co-workers/ close-friends/ neighbour)*

○ Yes (1)

○ No (2)

○ Refuse to answer (3)

Q38 Do you consume alcohol **regularly**? (*Regular= more than thrice a week)*

○ Yes (1)

○ No (2)

○ Refuse to answer (3)

End of Block: This section has questions on your exposure to smoke from tobacco or other drugs

Start of Block: Medical History and stress

Q39 **The next few questions are about your and your family's health history:**

Q40 Have you been under excessive stress lately?

- ○ Yes (1)
- ○ No (2)
- ○ Refuse to answer (3)

Q41 Does anyone in your family suffer from high blood pressure?
(parents, any grandparent, sibling, parents' sibling)

- ○ Yes (1)
- ○ No (2)
- ○ Refuse to answer (3)

Q42 Have you ever been diagnosed with hypertension (high blood pressure)?

- ○ Yes (1)
- ○ No (2)
- ○ Refuse to answer (3)

Q43 Are you: on medication for High Blood Pressure?

- ◯ Yes (1)
- ◯ No (2)
- ◯ Refuse to answer (3)

Q44 Do you suffer from any other long term illness? *(Other than high blood pressure*)

- ◯ Yes (1)
- ◯ No (2)
- ◯ Refuse to answer (3)

Skip To: End of Block If Do you suffer from any other long term illness? (Other than high blood pressure) = No

Skip To: End of Block If Do you suffer from any other long term illness? (Other than high blood pressure) = Refuse to answer

---

Q45 Please name the illness:

- ☐ Illness 1 (1) ____
- ☐ Illness 2 (2) ____
- ☐ Illness 3 (3) ____

Q46 Are you taking medication for the above ?

- ◯ Yes (1)
- ◯ No (2)

Q84 Section 1 of the survey ends here. The next section has questions regarding your neighbourhood.

End of Block: Medical History and stress

Start of Block: This module has questions regarding your perceptions of the neighborhood you cur

Q47 **This module has questions regarding <u>your perceptions of the neighborhood </u>you currently live in. It assesses the physical space, the facilities, and safety.**

Your neighbourhood is the walkable area around your home (roughly about a kilometres' radius around your home). Please answer the questions based on your living experience.

Q48 **The neighbourhood I live in:**

Q49 Offers me open space for a physically active lifestyle

- ◯ Yes (1)
- ◯ No (2)
- ◯ Don't Know (3)

Q50 Offers me open space for playing sports

- ◯ Yes (1)
- ◯ No (2)
- ◯ Don't Know (3)

Q51 Has cultural activities that I can be a part of

- Yes (1)
- No (2)
- Don't Know (3)

Q52 Has an event space I can visit for theatre, arts, and cultural immersion

- Yes (1)
- No (2)
- Don't Know (3)

Q53 Has a religious facility I can visit

- Yes (1)
- No (2)
- Don't Know (3)

Q54 Offers me public transport options for my need to connect within the city

- Yes (1)
- No (2)
- Don't Know (3)

Q55 Has a library that I can use

- Yes (1)
- No (2)
- Don't Know (3)

Q56 Has a school that my children can go to

- Yes (1)
- No (2)
- Don't Know/ Not Applicable (3)

Q57 Has a fresh food market that I can use

- Yes (1)
- No (2)
- Don't Know (3)

Q58 Has a fresh meat and fish market that I can use

- Yes (1)
- No (2)
- Don't Know (3)

Q59 Has a store I can buy groceries at

- ◯ Yes (1)
- ◯ No (2)
- ◯ Don't Know (3)

Q60 Has a health-care clinic I can go to

- ◯ Yes (1)
- ◯ No (2)
- ◯ Don't know (3)

Q61 Has a general/ departmental store where I can buy sundry items I often need

- ◯ Yes (1)
- ◯ No (2)
- ◯ Don't Know (3)

Q62 Has a good pedestrian path that I can use as I go about my daily activites

- ◯ Yes (1)
- ◯ No (2)
- ◯ Don't Know (3)

Q63 Has a community/ society gymnasium that I can access for fitness

- ◯ Yes (1)
- ◯ No (2)
- ◯ Don't Know (3)

Q64 Has a community/ society swimming pool I can access for fitness and leisure

- ◯ Yes (1)
- ◯ No (2)
- ◯ Don't Know (3)

Q65 Has a neighborhood/ community organization that I can be a part of

- ◯ Yes (1)
- ◯ No (2)
- ◯ Don't know (3)

End of Block: This module has questions regarding your perceptions of the neighborhood you cur

---

Start of Block: Neighborhood Questions continued

Q66 **The neighbourhood I live in:**

Q67 Offers children open space/ garden for playing

- Yes (1)
- No (2)
- Don't Know (3)

Q68 Is aesthetically pleasing (in other words, you perceive your neighbourhood as beautiful)

- Yes (1)
- No (2)
- Don't know (3)

Q69 Has a good pedestrian path that the elderly and physically challenged can use

- Yes (1)
- No (2)
- Don't Know (3)

Q70 Is a close-knit neighborhood where everyone knows each other and socializes

- Yes (1)
- No (2)
- Don't Know (3)

Q71 Has zebra crossings and regulated traffic and therefore is safe for crossing streets

- Yes (1)
- No (2)
- Don't Know (3)

Q72 Has corner stores for easy access to regular grocery needs

- Yes (1)
- No (2)
- Don't Know (3)

Q73 Has a 24 hour medical store/ pharmacy

- Yes (1)
- No (2)
- Don't Know (3)

Q74 Has a hospital for emergency and other medical needs that may arise

- Yes (1)
- No (2)
- Don't Know (3)

Q75 Is safe for individuals of **all gender** to be out and about

- ◯ Yes (1)
- ◯ No (2)
- ◯ Don't Know (3)

Q76 Is safe for individuals of **all ages** to be out and about

- ◯ Yes (1)
- ◯ No (2)
- ◯ Don't Know (3)

Q77 Is safe for individuals of **any socio-economic class** to be out and about

- ◯ Yes (1)
- ◯ No (2)
- ◯ Don't Know (3)

Q78 Is green with trees providing shade on footpaths

- ◯ Yes (1)
- ◯ No (2)
- ◯ Don't Know (3)

Q79 Is generally free from litter

◯ Yes (1)

◯ No (2)

◯ Don't Know (3)

Q80 Has green pockets, such as parks

◯ Yes (1)

◯ No (2)

◯ Don't Know (3)

End of Block: Neighborhood Questions continued

Start of Block: End of Survey, email information

Q81 You have finished the questionnaire. Thank you for your responses.

---

*

Q82 Please meet with Dr. Hitakshi Sehgal and she will record your blood pressure, height, and weight. The survey will then be submitted.

End of Block: End of Survey, email information

Start of Block: For Hitakshi Sehgal

Display This Question:

If If Please meet with Dr. Hitakshi Sehgal and she will record your blood pressure, height, and weight.... Text Response Is Equal to 8589

*

Q83 Blood Pressure, Height and Weight:

- ☐ Systolic Blood Pressure 1 (1) ____
- ☐ Diastolic Blood pressure 1 (2) ____
- ☐ Height (3) ____
- ☐ Weight (4) ____
- ☐ Birth Weight KG (5) ____
- ☐ Birth Weight Pounds (6) ____
- ☐ Systolic Blood Pressure 2 (7) ____
- ☐ Diastolic Blood Pressure 2 (8) ____
- ☐ StudyID (9) ____

Q87 Nature of Residency

- ○ Baug (1) ____
- ○ Parsi Apartment (2) ____
- ○ Dadar Parsee Colony (3) ____
- ○ Cosmopolitan (4) ____

End of Block: For Hitakshi Sehgal
